# Octa­aqua­bis(μ_2_-1*H*-pyrazole-3,5-di­carboxyl­ato)tricopper(II) tetra­hydrate

**DOI:** 10.1107/S1600536810001595

**Published:** 2010-01-30

**Authors:** Zhi-Gang Li, Shao-Ai Li, De-Quan Liu, Yi-Hua Huang, Jing-Wei Xu

**Affiliations:** a Shenzhen Environmental Monitoring Center, Shenzhen 518008, People’s Republic of China; b Shenzhen Environmental Protecting Bureau, Shenzhen 518008, People’s Republic of China; c State Key Laboratory of Electroanalytical Chemistry, Changchun Institute of Applied Chemistry, Chinese Academy of Sciences, Changchun 130022, People’s Republic of China

## Abstract

In the trinucler Cu^II^ complex mol­ecule of the title compound, [Cu_3_(C_5_HN_2_O_4_)_2_(H_2_O)_8_]·4H_2_O, the central Cu^II^ atom is located on an inversion centre and is coordinated in a distorted octa­hedral geometry. The equatorial sites are occupied by two N and two O atoms from two pyrazole-3,5-dicarboxyl­ate ligands and the axial positions are occupied by two water mol­ecules. The two other symmetry-related Cu^II^ atoms are penta­coordinated and assume a square-pyramidal geometry. In the crystal structure, coordinated and uncoordinated water mol­ecules and carboxyl­ate O atoms are linked by O—H⋯O hydrogen bonds.

## Related literature

For general background to coordination polymers, see: Yaghi *et al.* (2003 ▶); Kitagawa *et al.* (2004 ▶). For related structures, see: King *et al.* (2003 ▶); Li (2005 ▶). For graph-set motifs, see: Bernstein *et al.* (1995 ▶).
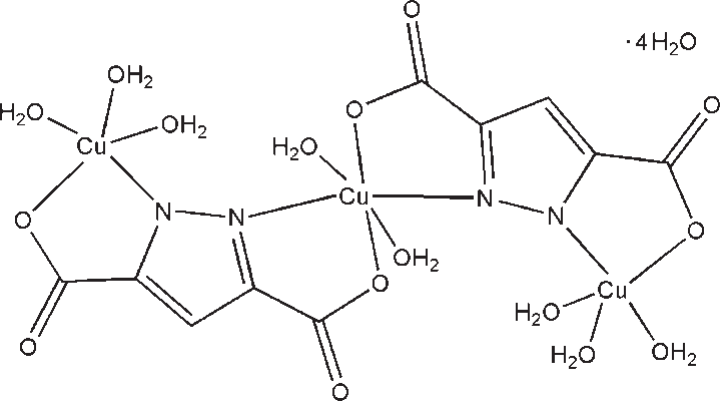


## Experimental

### 

#### Crystal data

[Cu_3_(C_5_HN_2_O_4_)_2_(H_2_O)_8_]·4H_2_O
*M* *_r_* = 712.97
Triclinic, 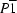

*a* = 8.9455 (6) Å
*b* = 9.1018 (7) Å
*c* = 9.1125 (7) Å
α = 103.485 (1)°
β = 90.924 (1)°
γ = 117.505 (1)°
*V* = 633.31 (8) Å^3^
*Z* = 1
Mo *K*α radiation
μ = 2.59 mm^−1^
*T* = 293 K
0.17 × 0.13 × 0.05 mm

#### Data collection

Bruker SMART APEX CCD area-detector diffractometer
Absorption correction: multi-scan (*SADABS*; Sheldrick, 1996 ▶) *T*_min_ = 0.666, *T*_max_ = 0.877
3535 measured reflections
2412 independent reflections
2198 reflections with *I* > 2σ(*I*)
*R*_int_ = 0.010

#### Refinement

*R*[*F* ^2^ > 2σ(*F* ^2^)] = 0.036
*wR*(*F* ^2^) = 0.095
*S* = 1.08
2412 reflections
205 parameters
12 restraints
H atoms treated by a mixture of independent and constrained refinement
Δρ_max_ = 0.89 e Å^−3^
Δρ_min_ = −0.54 e Å^−3^

### 

Data collection: *SMART* (Bruker, 1998 ▶); cell refinement: *SAINT-Plus* (Bruker, 1998 ▶); data reduction: *SAINT-Plus*; program(s) used to solve structure: *SHELXS97* (Sheldrick, 2008 ▶); program(s) used to refine structure: *SHELXL97* (Sheldrick, 2008 ▶); molecular graphics: *SHELXTL* (Sheldrick, 2008 ▶); software used to prepare material for publication: *SHELXTL*.

## Supplementary Material

Crystal structure: contains datablocks global, I. DOI: 10.1107/S1600536810001595/is2499sup1.cif

Structure factors: contains datablocks I. DOI: 10.1107/S1600536810001595/is2499Isup2.hkl

Additional supplementary materials:  crystallographic information; 3D view; checkCIF report

## Figures and Tables

**Table 1:** Hydrogen-bond geometry (Å, °)

*D*—H⋯*A*	*D*—H	H⋯*A*	*D*⋯*A*	*D*—H⋯*A*
O5—H5*A*⋯O1^i^	0.87 (3)	2.28 (4)	3.050 (4)	148 (4)
O5—H5*B*⋯O8^ii^	0.87 (4)	2.16 (4)	3.021 (5)	172 (4)
O6—H6*A*⋯O8^iii^	0.87 (4)	2.38 (5)	3.078 (4)	137 (4)
O6—H6*B*⋯O2^iii^	0.87 (6)	2.30 (6)	3.077 (4)	149 (4)
O7—H7*A*⋯O1^ii^	0.80 (4)	2.16 (4)	2.860 (4)	147 (4)
O7—H7*B*⋯O4^iv^	0.81 (4)	1.91 (4)	2.715 (4)	171 (4)
O8—H8*A*⋯O7^v^	0.81 (3)	2.06 (3)	2.854 (4)	169 (4)
O8—H8*B*⋯O1	0.81 (5)	2.03 (5)	2.836 (4)	175 (4)
O9—H9*A*⋯O4^vi^	0.87 (4)	2.26 (4)	3.061 (4)	154 (4)

Symmetry codes: (i) 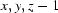; (ii) 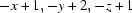; (iii) 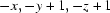; (iv) 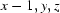; (v) 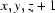; (vi) 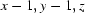.

**Fig. 1.**
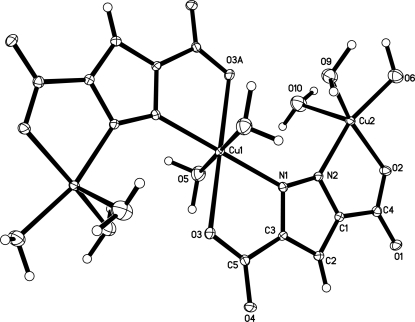
A view of (I), with the atom-labeling scheme and 30% probability displacement ellipsoids. [Symmetry code: (A) 1 - x, 1 - y, -z.]

**Fig. 2.**
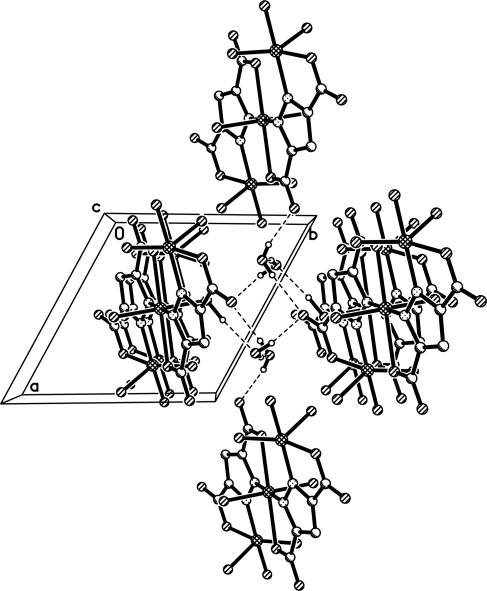
Perspective view of packing structure of (I) along the c axis. For the sake of clarity, H atoms not involved in hydrogen bonds have been omitted.

## supplementary crystallographic information

### Comment

The design and synthesis of novel coordination architectures is a fertile field due to the intriguing network topologies and potential a pplications as new classes of materials (Yaghi *et al.*, 2003; Kitagawa *et al.*, 2004). The ligand of pyrazole-3,5-dicarboxylic acid has several potential coordination sites involving both two N atoms of the pyrazole ring and four carboxylate O atoms. These multifunctional coordination sites are highly accessible to metal ions, as such, the ligand can coordinate as a mono-, bi-, or tetradentate ligand and can act to link together metal centers through a number of bridging modes (Li, 2005). The divalent copper atoms are easily to precipitate with the OH^-^ when the pyrazole-3,5-dicarboxylic acids are deprotoned in base water solution, the mixed solution can obtain coordianted polymer single crystals in hydrothermal condition (King *et al.*, 2003). Nevertheless, when the ammonia was added to the mixed solution, because of the complexing action between the copper atoms and NH_3_, the turbid soltuion became clear. After the ammonia slowly evaporated, we obtained the blue crystals, compound (I), as shown in Fig.1, a copper(II) trimer.

The central copper atom, Cu1, lies on a crystallographic inversion center. The Cu1 atom has a six-coordinate octahedral geometry, in which two O atoms and two N atoms from two pyrazole-3,5-dicarboxylate ligands occupy the equatorial plane, and the axial coordination sites are occupied two water molecules; the Cu—N/O bond distances range from 2.003 (2) to 2.437 (3) Å. The other two symmetry-related copper atoms, Cu2, have a pentacoordinate square-pyramidal geometry, where a pyrazole nitrogen N2 and a carboxylate oxygen O3 from one pyrazole-3,5-dicarboxylate ligand occupy two coordination sites and the remaining three positions are occupied by water molecules; the Cu—N/O bond distances range from 1.984 (2) to 2.237 (2) Å. The pyrazole-3,5-dicarboxylate ligand is not strictly planar. Deviation from the mean plane defined by the pyrazole ring is seen for both carboxylate groups with values ranging from 0.034 (1) to 0.205 (1) Å. The dihedral angle between the two carboxylate mean planes is 11.3 (3)°. It can be seen that the ligand bite angle at the two different copper centers Cu1 and Cu2 is similar, 74.8 (4) and 80.6 (4)°, respectively. This implies that the pyrazole-3,5-dicarboxylate ligand is a fairly rigid ligand and retains its integrity on metal chelation.

In the asymmetric unit, there are two lattice water molecules, four coordinated water molecules and carboxylate O atoms, which form complexed hydrogen-bonding interactions. Two lattice water molecules and its symmetric equivalents together with two carboxylate O atoms from two trimers form a hydrogen-bonded chair conformation, generating an *R*_4_^6^(6) motif (Bernstein *et al.*, 1995). Meanwhile, the four lattice water molecules in each *R*_4_^6^(6) motif also bind four another trimers by O7—H7B···O4 hydrogen bond interaction, and O5—H5B···O8 hydrogen bond interaction. Those strong hydrogen-bonding interactions as well as some weaker interactions, such as O5—H5A···O1, O6—H6A···O8, O6—H6B···O2 and O9—H9A···O4, extend the crystal structure into a three-dimensional supramolecular network (Fig. 2).

### Experimental

The title complex was prepared by the addition of Cu(BF_4_)_2_ (20 mmol) and pyrazole-3,5-dicarboxylic acid (30 mmol) to 40 ml water. The mixture was stirred for 1 h, a blue precipitate was obtained. A minimum amount of ammonia (14 *M*) was added to give a blue solution. Suitable crystals were obtained after standing at room temperature for several days (yield 51% based on Cu).

### Refinement

Atom H2 was placed geometrically  (C—H = 0.93 Å) and refined using a riding model, with *U*_iso_(H) = *U*_eq_(C). The H atoms bonded to O atoms of water molecules were located in a difference Fourier map and refined, with a bond distance restriction [O—H = 0.82 (2) Å], and with *U*_iso_(H) = 1.2*U*_eq_(O).

### Crystal data

**Table:**

[Cu_3_(C_5_HN_2_O_4_)_2_(H_2_O)_8_]·4H_2_O	*Z* = 1
*M**_r_* = 712.97	*F*(000) = 361
Triclinic, *P*1	*D*_x_ = 1.869 Mg m^−^^3^
Hall symbol: -P 1	Mo *K*α radiation, λ = 0.71073 Å
*a* = 8.9455 (6) Å	Cell parameters from 2128 reflections
*b* = 9.1018 (7) Å	θ = 2.3–26.0°
*c* = 9.1125 (7) Å	µ = 2.59 mm^−^^1^
α = 103.485 (1)°	*T* = 293 K
β = 90.924 (1)°	Tabular, blue
γ = 117.505 (1)°	0.17 × 0.13 × 0.05 mm
*V* = 633.31 (8) Å^3^	

### Data collection

**Table:**

Bruker SMART APEX CCD area-detector diffractometer	2412 independent reflections
Radiation source: fine-focus sealed tube	2198 reflections with *I* > 2σ(*I*)
graphite	*R*_int_ = 0.010
φ and ω scans	θ_max_ = 26.0°, θ_min_ = 2.3°
Absorption correction: multi-scan (*SADABS*; Sheldrick, 1996)	*h* = −10→11
*T*_min_ = 0.666, *T*_max_ = 0.877	*k* = −11→9
3535 measured reflections	*l* = −11→9

### Refinement

**Table:**

Refinement on *F*^2^	Primary atom site location: structure-invariant direct methods
Least-squares matrix: full	Secondary atom site location: difference Fourier map
*R*[*F*^2^ > 2σ(*F*^2^)] = 0.036	Hydrogen site location: inferred from neighbouring sites
*wR*(*F*^2^) = 0.095	H atoms treated by a mixture of independent and constrained refinement
*S* = 1.08	*w* = 1/[σ^2^(*F*_o_^2^) + (0.0495*P*)^2^ + 1.615*P*] where *P* = (*F*_o_^2^ + 2*F*_c_^2^)/3
2412 reflections	(Δ/σ)_max_ = 0.042
205 parameters	Δρ_max_ = 0.89 e Å^−^^3^
12 restraints	Δρ_min_ = −0.54 e Å^−^^3^

### Special details

**Table:**

Geometry. All e.s.d.'s (except the e.s.d. in the dihedral angle between two l.s. planes) are estimated using the full covariance matrix. The cell e.s.d.'s are taken into account individually in the estimation of e.s.d.'s in distances, angles and torsion angles; correlations between e.s.d.'s in cell parameters are only used when they are defined by crystal symmetry. An approximate (isotropic) treatment of cell e.s.d.'s is used for estimating e.s.d.'s involving l.s. planes.
Refinement. Refinement of *F*^2^ against ALL reflections. The weighted *R*-factor *wR* and goodness of fit *S* are based on *F*^2^, conventional *R*-factors *R* are based on *F*, with *F* set to zero for negative *F*^2^. The threshold expression of *F*^2^ > σ(*F*^2^) is used only for calculating *R*-factors(gt) *etc*. and is not relevant to the choice of reflections for refinement. *R*-factors based on *F*^2^ are statistically about twice as large as those based on *F*, and *R*- factors based on ALL data will be even larger.

### Fractional atomic coordinates and isotropic or equivalent isotropic displacement parameters (Å^2^)

**Table:**

	*x*	*y*	*z*	*U*_iso_*/*U*_eq_	
Cu1	0.5000	0.5000	0.0000	0.01250 (16)	
Cu2	0.18693 (5)	0.39943 (5)	0.35269 (4)	0.01365 (14)	
O1	0.4661 (3)	0.8149 (3)	0.7106 (3)	0.0205 (5)	
O2	0.2463 (3)	0.5801 (3)	0.5590 (3)	0.0180 (5)	
O3	0.8061 (3)	0.6833 (3)	0.0762 (3)	0.0187 (5)	
O4	0.9786 (3)	0.8716 (3)	0.2903 (3)	0.0198 (5)	
O5	0.4555 (4)	0.6959 (4)	−0.0015 (3)	0.0319 (7)	
H5A	0.420 (6)	0.690 (7)	−0.093 (3)	0.038*	
H5B	0.541 (5)	0.797 (4)	0.040 (5)	0.038*	
O6	−0.0225 (4)	0.2439 (4)	0.4269 (4)	0.0318 (7)	
H6A	−0.091 (5)	0.148 (4)	0.360 (5)	0.038*	
H6B	−0.083 (6)	0.291 (6)	0.468 (5)	0.038*	
O7	0.2443 (4)	0.8545 (4)	0.1630 (3)	0.0268 (6)	
H7A	0.330 (4)	0.923 (5)	0.219 (5)	0.032*	
H7B	0.159 (4)	0.848 (6)	0.198 (5)	0.032*	
O8	0.2631 (4)	0.9378 (4)	0.8781 (3)	0.0280 (6)	
H8A	0.245 (6)	0.913 (6)	0.958 (3)	0.034*	
H8B	0.316 (5)	0.899 (6)	0.826 (5)	0.034*	
O9	0.1851 (4)	0.2020 (4)	0.1939 (4)	0.0335 (7)	
H9A	0.104 (5)	0.102 (4)	0.195 (6)	0.040*	
H9B	0.282 (4)	0.202 (7)	0.197 (6)	0.040*	
O10	0.0459 (4)	0.4770 (5)	0.2092 (4)	0.0415 (8)	
H10A	0.102 (6)	0.580 (4)	0.202 (6)	0.050*	
H10B	−0.052 (4)	0.452 (7)	0.238 (6)	0.050*	
N1	0.5326 (3)	0.5804 (4)	0.2330 (3)	0.0135 (6)	
N2	0.4214 (3)	0.5670 (4)	0.3334 (3)	0.0136 (6)	
C1	0.5045 (4)	0.6944 (4)	0.4637 (4)	0.0136 (6)	
C2	0.6742 (4)	0.7934 (4)	0.4484 (4)	0.0151 (7)	
H2	0.7597	0.8884	0.5196	0.015*	
C3	0.6852 (4)	0.7162 (4)	0.3012 (4)	0.0125 (6)	
C4	0.4011 (4)	0.6993 (4)	0.5882 (4)	0.0153 (7)	
C5	0.8344 (4)	0.7607 (4)	0.2152 (4)	0.0136 (7)	

### Atomic displacement parameters (Å^2^)

**Table:**

	*U*^11^	*U*^22^	*U*^33^	*U*^12^	*U*^13^	*U*^23^
Cu1	0.0145 (3)	0.0118 (3)	0.0090 (3)	0.0055 (2)	0.0011 (2)	0.0007 (2)
Cu2	0.0111 (2)	0.0123 (2)	0.0137 (2)	0.00298 (17)	0.00279 (15)	0.00235 (16)
O1	0.0194 (12)	0.0210 (13)	0.0134 (12)	0.0060 (11)	0.0028 (10)	−0.0012 (10)
O2	0.0141 (12)	0.0167 (12)	0.0181 (12)	0.0045 (10)	0.0046 (9)	0.0018 (10)
O3	0.0171 (12)	0.0216 (13)	0.0122 (12)	0.0056 (10)	0.0040 (9)	0.0030 (10)
O4	0.0116 (11)	0.0210 (13)	0.0172 (12)	0.0012 (10)	0.0018 (9)	0.0026 (10)
O5	0.0366 (17)	0.0299 (16)	0.0294 (16)	0.0160 (14)	0.0037 (13)	0.0082 (13)
O6	0.0251 (15)	0.0303 (16)	0.0354 (17)	0.0099 (13)	0.0063 (12)	0.0076 (13)
O7	0.0209 (14)	0.0209 (14)	0.0335 (16)	0.0080 (12)	0.0098 (12)	0.0025 (12)
O8	0.0286 (15)	0.0294 (16)	0.0257 (15)	0.0154 (13)	0.0053 (12)	0.0033 (12)
O9	0.0323 (16)	0.0270 (16)	0.0358 (17)	0.0100 (13)	0.0082 (13)	0.0076 (13)
O10	0.0351 (18)	0.0381 (19)	0.054 (2)	0.0156 (16)	0.0024 (16)	0.0220 (16)
N1	0.0125 (13)	0.0140 (14)	0.0123 (13)	0.0050 (11)	0.0035 (10)	0.0035 (11)
N2	0.0119 (13)	0.0146 (14)	0.0112 (13)	0.0044 (11)	0.0020 (10)	0.0018 (11)
C1	0.0136 (15)	0.0139 (16)	0.0119 (15)	0.0057 (13)	0.0014 (12)	0.0032 (12)
C2	0.0125 (15)	0.0157 (17)	0.0132 (16)	0.0039 (13)	0.0005 (12)	0.0031 (13)
C3	0.0127 (15)	0.0126 (16)	0.0103 (15)	0.0047 (13)	−0.0010 (12)	0.0027 (12)
C4	0.0149 (16)	0.0166 (17)	0.0152 (16)	0.0081 (14)	0.0031 (13)	0.0047 (13)
C5	0.0150 (16)	0.0130 (17)	0.0133 (16)	0.0064 (14)	0.0037 (13)	0.0048 (13)

### Geometric parameters (Å, °)

**Table:**

Cu1—O5	2.001 (3)	O6—H6B	0.87 (6)
Cu1—O5^i^	2.001 (3)	O7—H7A	0.80 (2)
Cu1—N1	2.047 (3)	O7—H7B	0.81 (2)
Cu1—N1^i^	2.047 (3)	O8—H8A	0.81 (2)
Cu1—O3^i^	2.437 (2)	O8—H8B	0.81 (5)
Cu1—O3	2.437 (2)	O9—H9A	0.87 (2)
Cu2—N2	1.985 (3)	O9—H9B	0.87 (5)
Cu2—O6	2.002 (3)	O10—H10A	0.85 (2)
Cu2—O9	2.021 (3)	O10—H10B	0.86 (5)
Cu2—O2	2.059 (2)	N1—N2	1.345 (4)
Cu2—O10	2.236 (3)	N1—C3	1.354 (4)
O1—C4	1.247 (4)	N2—C1	1.357 (4)
O2—C4	1.277 (4)	C1—C2	1.392 (5)
O3—C5	1.255 (4)	C1—C4	1.481 (5)
O4—C5	1.264 (4)	C2—C3	1.390 (5)
O5—H5A	0.87 (2)	C2—H2	0.9300
O5—H5B	0.87 (2)	C3—C5	1.497 (4)
O6—H6A	0.87 (2)		
			
O5—Cu1—O5^i^	180.0	Cu2—O6—H6B	116 (3)
O5—Cu1—N1	87.36 (12)	H6A—O6—H6B	108 (5)
O5^i^—Cu1—N1	92.64 (12)	H7A—O7—H7B	113 (5)
O5—Cu1—N1^i^	92.64 (12)	H8A—O8—H8B	117 (5)
O5^i^—Cu1—N1^i^	87.36 (12)	Cu2—O9—H9A	114 (3)
N1—Cu1—N1^i^	180.00 (18)	Cu2—O9—H9B	114 (3)
O5—Cu1—O3^i^	85.89 (11)	H9A—O9—H9B	110 (5)
O5^i^—Cu1—O3^i^	94.11 (11)	Cu2—O10—H10A	115 (4)
N1—Cu1—O3^i^	105.11 (9)	Cu2—O10—H10B	109 (4)
N1^i^—Cu1—O3^i^	74.89 (9)	H10A—O10—H10B	114 (6)
O5—Cu1—O3	94.11 (11)	N2—N1—C3	107.6 (3)
O5^i^—Cu1—O3	85.89 (11)	N2—N1—Cu1	132.2 (2)
N1—Cu1—O3	74.89 (9)	C3—N1—Cu1	116.3 (2)
N1^i^—Cu1—O3	105.11 (9)	N1—N2—C1	108.4 (3)
O3^i^—Cu1—O3	180.0	N1—N2—Cu2	137.8 (2)
N2—Cu2—O6	165.29 (12)	C1—N2—Cu2	113.2 (2)
N2—Cu2—O9	93.98 (12)	N2—C1—C2	110.0 (3)
O6—Cu2—O9	92.94 (13)	N2—C1—C4	115.9 (3)
N2—Cu2—O2	80.69 (10)	C2—C1—C4	134.1 (3)
O6—Cu2—O2	88.18 (11)	C3—C2—C1	103.4 (3)
O9—Cu2—O2	158.66 (12)	C3—C2—H2	128.3
N2—Cu2—O10	97.78 (12)	C1—C2—H2	128.3
O6—Cu2—O10	93.87 (13)	N1—C3—C2	110.6 (3)
O9—Cu2—O10	99.41 (14)	N1—C3—C5	119.3 (3)
O2—Cu2—O10	101.78 (12)	C2—C3—C5	130.1 (3)
C4—O2—Cu2	114.3 (2)	O1—C4—O2	124.6 (3)
C5—O3—Cu1	109.0 (2)	O1—C4—C1	120.1 (3)
Cu1—O5—H5A	111 (3)	O2—C4—C1	115.2 (3)
Cu1—O5—H5B	115 (3)	O3—C5—O4	125.8 (3)
H5A—O5—H5B	110 (5)	O3—C5—C3	117.4 (3)
Cu2—O6—H6A	115 (3)	O4—C5—C3	116.8 (3)

Symmetry codes: (i) −*x*+1, −*y*+1, −*z*.

### Hydrogen-bond geometry (Å, °)

**Table:**

*D*—H···*A*	*D*—H	H···*A*	*D*···*A*	*D*—H···*A*
O5—H5A···O1^ii^	0.87 (3)	2.28 (4)	3.050 (4)	148 (4)
O5—H5B···O8^iii^	0.87 (4)	2.16 (4)	3.021 (5)	172 (4)
O6—H6A···O8^iv^	0.87 (4)	2.38 (5)	3.078 (4)	137 (4)
O6—H6B···O2^iv^	0.87 (6)	2.30 (6)	3.077 (4)	149 (4)
O7—H7A···O1^iii^	0.80 (4)	2.16 (4)	2.860 (4)	147 (4)
O7—H7B···O4^v^	0.81 (4)	1.91 (4)	2.715 (4)	171 (4)
O8—H8A···O7^vi^	0.81 (3)	2.06 (3)	2.854 (4)	169 (4)
O8—H8B···O1	0.81 (5)	2.03 (5)	2.836 (4)	175 (4)
O9—H9A···O4^vii^	0.87 (4)	2.26 (4)	3.061 (4)	154 (4)

Symmetry codes: (ii) *x*, *y*, *z*−1; (iii) −*x*+1, −*y*+2, −*z*+1; (iv) −*x*, −*y*+1, −*z*+1; (v) *x*−1, *y*, *z*; (vi) *x*, *y*, *z*+1;  (vii) *x*−1, *y*−1, *z*.
